# Expanding and Enriching the LncRNA Gene–Disease Landscape Using the GeneCaRNA Database

**DOI:** 10.3390/biomedicines12061305

**Published:** 2024-06-12

**Authors:** Shalini Aggarwal, Chana Rosenblum, Marshall Gould, Shahar Ziman, Ruth Barshir, Ofer Zelig, Yaron Guan-Golan, Tsippi Iny-Stein, Marilyn Safran, Shmuel Pietrokovski, Doron Lancet

**Affiliations:** 1 Department of Molecular Genetics, Weizmann Institute of Science, Herzl 234, Rehovot 7610010, Israelshahar.ziman@weizmann.ac.il (S.Z.);; 2 Department of Biological Sciences, University College London, Gower Street, London WC1E 6BT, UK; 3 TAD Center for AI and Data Science, Tel Aviv University, Tel Aviv 6997801, Israel; 4 LifeMap Sciences Inc., Alameda, CA 94501, USA

**Keywords:** non-coding RNA, long non-coding RNA, human noncoding RNA, ncRNA, lncRNA, subclassification, GeneCards, GeneCaRNA

## Abstract

The GeneCaRNA human gene database is a member of the GeneCards Suite. It presents ~280,000 human non-coding RNA genes, identified algorithmically from ~690,000 RNAcentral transcripts. This expands by ~tenfold the ncRNA gene count relative to other sources. GeneCaRNA thus contains ~120,000 long non-coding RNAs (LncRNAs, >200 bases long), including ~100,000 novel genes. The latter have sparse functional information, a vast terra incognita for future research. LncRNA genes are uniformly represented on all nuclear chromosomes, with 10 genes on mitochondrial DNA. Data obtained from MalaCards, another GeneCards Suite member, finds 1547 genes associated with 1 to 50 diseases. About 15% of the associations portray experimental evidence, with cancers tending to be multigenic. Preliminary text mining within GeneCaRNA discovers interactions of lncRNA transcripts with target gene products, with 25% being ncRNAs and 75% proteins. GeneCaRNA has a biological pathways section, which at present shows 131 pathways for 38 lncRNA genes, a basis for future expansion. Finally, our GeneHancer database provides regulatory elements for ~110,000 lncRNA genes, offering pointers for co-regulated genes and genetic linkages from enhancers to diseases. We anticipate that the broad vista provided by GeneCaRNA will serve as an essential guide for further lncRNA research in disease decipherment.

## 1. Introduction

As our understanding of the human genome deepens, the spotlight on non-coding RNAs (ncRNAs) has intensified, revealing a diverse landscape of non-coding transcripts. Currently, the major sources (HGNC [1], NCBI Gene [2], Ensembl [3], and RNAcentral [4]), portray ~38,000 well-documented ncRNA genes. In our earlier paper [5], we significantly augmented the count of identified ncRNA genes to 220,000, obtained based on algorithms that analyzed all transcripts in RNAcentral [4]. Our ncRNA gene category consists of 24 classes, with the highest counts belonging to lncRNAs, piRNAs, and miRNAs. 

GeneCaRNA’s *raison d’etre* within the GeneCards Suite is to provide a comprehensive gene-centric knowledge base for human ncRNA genes and their annotations. The present version 5.19 GeneCaRNA exhibits ~280,000 human non-coding RNA genes, stemming from ~690,000 RNAcentral transcripts. A key advancement in GeneCaRNA is the addition of a very large number of potentially functional ncRNA genes to a gene database that enables the use of a broad range of other types of genomic and biological data and offers many bioinformatic analysis tools.

GeneCaRNA is a multi-source database, offering a promising avenue to enrich annotation information, and functions on a single platform. GeneCaRNA effectively addresses the challenge of disparate gene names across sources by leveraging aliases, facilitating accurate gene mapping, and providing comprehensive gene details. GeneCaRNA is frequently updated to address novel knowledge and automatically add functional annotation and disease links which also address the novel Transcripts Inferred GeneCaRNA genes (TRIGGs) [5].

LncRNA genes constitute the largest class within the ncRNA gene category, ~46% of the total. This gene class is defined for transcripts longer than 200 nucleotides, which have scant or no protein-coding characteristics [6,7]. The large size of the lncRNA class led to further definition of subclasses, based on criteria such as transcriptional machinery [8], structure [9], expression regulation [10], location with respect to protein-coding genes [11,12], and binding targets [10], as well as transcriptional and post-transcriptional regulation [12]. 

LncRNA genes and their products are back-end regulators of the protein-coding genes. These genes are majorly known to contribute to cancer pathobiology [13] and also to some hereditary diseases [14]. The mechanism of action is poorly understood, and their low transcription level makes them a covert disease driver [15]. Their role in non-cancerous diseases is yet to be discovered. We make some headway in this direction using GeneCaRNA and MalaCards by shedding light on the potential relations between lncRNAs and non-cancerous diseases.

This paper describes thorough analyses of the lncRNA class of human genes and their role in deciphering pathogenesis based on the power of the GeneCards Suite. GeneCaRNA provides textual analyses on annotations of each lncRNA GeneCard in the database and reveals information for items such as revised subclassification, interactions between gene products (protein and transcripts), gene-to-disease relationships, and relations of genes to biological pathways. GeneCaRNA is poised to become a highly useful platform for accessing complete profiles of lncRNAs. 

## 2. Methods

### 2.1. Data Extraction and Text Mining 

Data extraction from the GeneCaRNA database (GC V5.19, based on RNAcentral V23) employed Structured Query Language (SQL) or our in-house tool, GeneAlaCart [16]. For routine analysis, we generated an interim lncRNA table containing the gene symbol, gene description, mined data sources, GeneCards Inferred Functionality Scores (GIFtS) [17], and chromosomal location of each gene (Table S1). 

The extracted table contained 120,982 lncRNA genes, whose categorization was based on transcript categorization in our sources. Some genes are assigned symbols based on the information in our major sources, and if absent there, are assigned our own GeneCards symbols, as previously assigned [16]. Most genes have additional (non-symbol) alias names featured in various external sources, all of which are included in GeneCaRNA. GeneCaRNA was also used to extract all of the aliases and protein existence (PE) [18], using GeneAlaCart. 

### 2.2. LncRNA Subclass Definition 

We extracted keywords from the Aliases section of GeneCaRNA, which contains multi-word gene descriptions. In the first stage, a list of the 10 most frequent words appearing in the entire collection of lncRNA genes was identified using in-house Python code (see Method S1 in Supplementary Materials). These terms were “Intronic”, “Intergenic”, “LINC”, “Overlapping”, “Divergent”, “Pseudogene”, “Antisense”, “Sense”, “Open Reading Frame”, and “ORF”. In the second stage, 5 candidate subclassification terms were chosen, consulting other subclassification approaches [3,11,19]. We performed sub-family term unification, e.g., “Overlapping”, “Pseudogene”, “Sense”, and “ORF”, which were clustered in our proposed system as “Protein Suspect”. Under the same subclass, we also joined genes with the highest degree (1, 2, 3) of UniProt’s PE Levels [18], and the genes with lower levels (4, 5) were also included provided that “protein” was a keyword appearing in our keyword search in GeneCaRNA’s Aliases section. Finally, each gene was assigned one or more subclasses, based on the appearance of these keywords in the original gene/alias-specific text in the Aliases section of GeneCaRNA. 

### 2.3. Gene–Disease and Gene-Pathway Associations

A list of diseases associated with each gene was obtained by an SQL query. Each gene–disease pairing was assigned an association score [16]. The score value depends on the level of manual curation of the information source, and on the significance assigned by the source itself to its different annotation classes. In addition, an “elite” grade is assigned for associations with experimental evidence support, and an “inferred” grade for the rest, where evidence is derived only from text-mining. The GeneCards policy is to also portray inferred associations to catalyze future experimentation. 

For gene-pathway relationships, we included only lncRNA genes with at least one associated disease. These were extracted using GeneAlaCart applied to the Pathways section of GeneCaRNA. The latter is mined from WikiPathways, SIGNOR, Reactome, PharmGKB, and Sino Biological pathways. In GeneCaRNA, we also show amalgamations to our SuperPathways facility [20]. 

### 2.4. Gene Product Interactions

The interaction targets for each lncRNA transcript (proteins, ncRNAs, or DNA segments) were identified by the Multigene Search (MuSe) in-house text mining program. The query gene can be a symbol or any of the aliases. This allows one to receive target genes for a long list of submitted genes, e.g., by submitting strings of the form “<gene name> binds”, or “<gene name> interacts with”. The output is texts from, e.g., the GeneCaRNA Publications or Summaries sections, which include the text query. The output file of the MuSe is further curated using in-house Python code (see Method S2 in Supplementary Materials).

## 3. Results

### 3.1. Data Extraction and Text Mining

**Figure 1:**
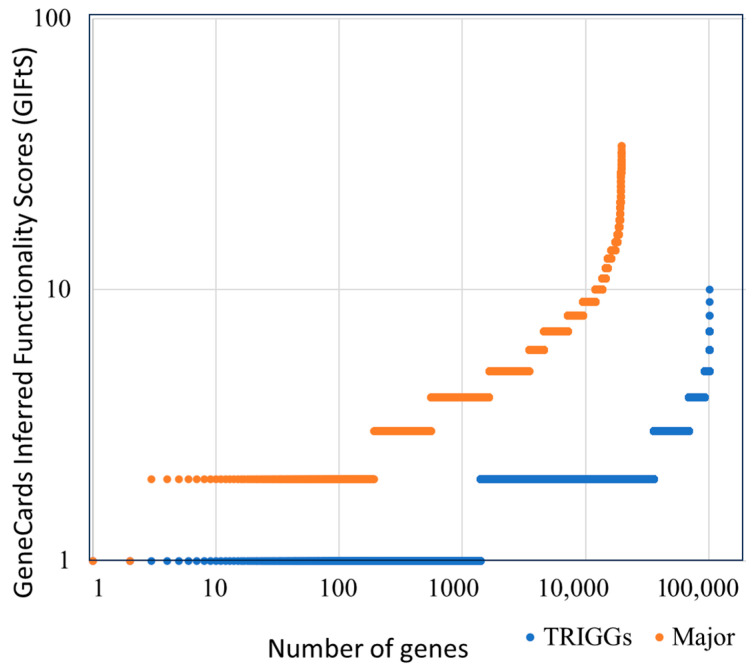
Rank graph for annotative information, separately illustrated for two groups: genes from the major gene sources (NCBI, HGNC, and ENSEMBL) (orange), and genes inferred from RNAcentral transcripts without any annotation from the major gene sources, TRIGGS (blue).

We extracted 120,982 lncRNA genes from GeneCaRNA, as portrayed in Table S1. Of these, 19,482 genes are available in the major sources, and 101,500 genes are unique GeneCaRNA TRIGGs. Both categories were explored for annotative information based on their GIFtS value, which represents the cumulative information imported from all the relevant sources [17]. We found that while 34% of the major source genes had GIFtS > 10, none of the TRIGGs had a GIFtS > 10 (Figure 1). This indicates a fertile area for future research in the realm of TRIGGs, both for lncRNA genes and other ncRNA categories.

### 3.2. LncRNA Subclass Definition 

**Figure 2:**
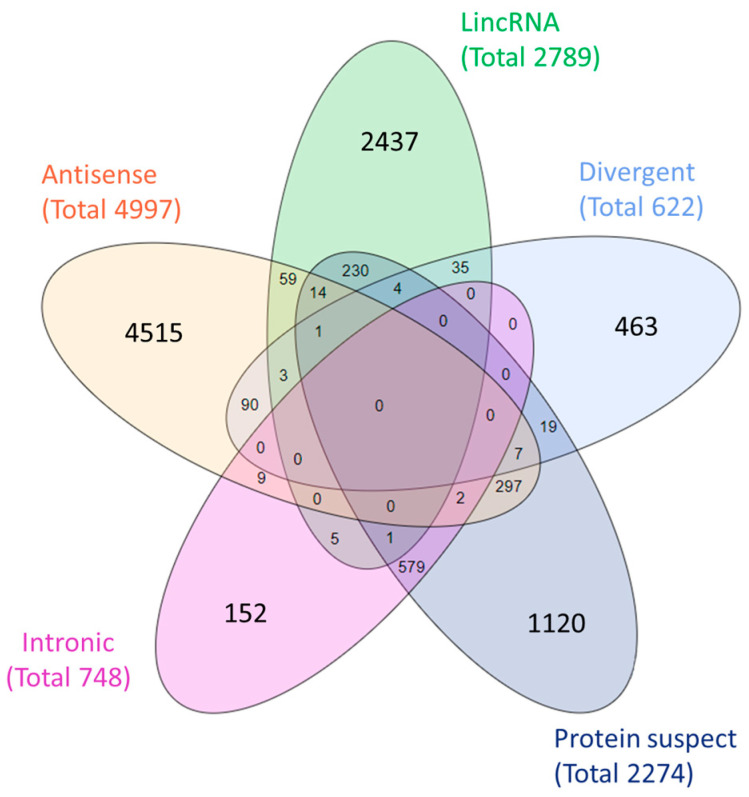
A GeneCaRNA-based suggested subclassification of 11,430 lncRNA subclass-definable genes (9.4% of the total). LincRNA: LncRNAs that are transcribed from the DNA stretch between the two protein-coding genes. Divergent: LncRNAs that are transcribed by a promoter shared with a protein-coding gene. Protein suspect: LncRNAs that have the potential to encode a peptide/protein. Intronic: LncRNAs transcribed purely from the intron(s) of a coding gene. Antisense: LncRNAs that are transcribed in antisense to a protein-coding DNA strand.

We developed a pipeline for defining subclasses for lncRNA genes (see Section 2). [11,19]. We performed sub-family term unification (see Section 2). As a result, five subclasses were created: “Antisense”, “LincRNA”, “Divergent”, “Protein Suspect”, and “Intronic” (Table S2). These subclasses cover only 9.4% of the relevant genes; the rest were classified as “no subclass” (Figure 2). 

### 3.3. Gene–Disease and Gene-Pathway Associations

**Figure 3:**
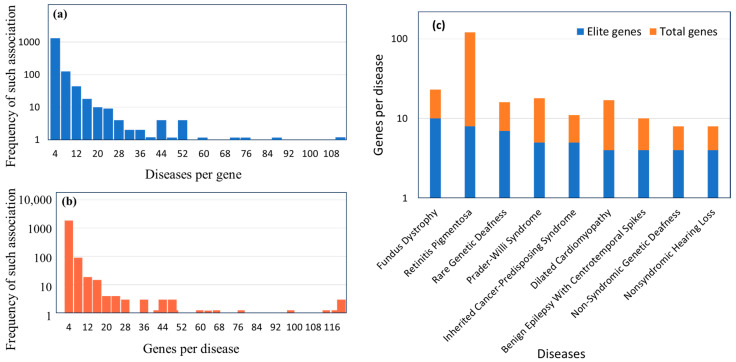
Gene–disease associations: Detailed scrutiny of the gene–disease associations among the 1554 lncRNA genes and 2019 diseases. (**a**) The count of diseases per gene values. (**b**) The count of genes per disease values. (**c**) The number of genes per disease for a sample of 9 diseases with 4 or more elite associations.

We systematically explored GeneCaRNA for relationships of lncRNA genes to diseases, as portrayed in MalaCards, the disease database of the GeneCards suite [21]. A total of 5100 gene–disease associations were found, spanning 1554 lncRNA genes and 2019 diseases. We identified 715 elite associations, involving 618 contributing diseases, and 340 contributing genes (Table S3). Among the 1554 genes, 793 were associated with only one disease, and the rest had two to one hundred eleven associated diseases (Figure 3a). The top five disease-contributing genes are H19 (111 diseases), MALAT1 (87 diseases), MEG3 (76 diseases), HOTAIR (69 diseases), and CDKN2B-AS1 (59 diseases). 

**Figure 4:**
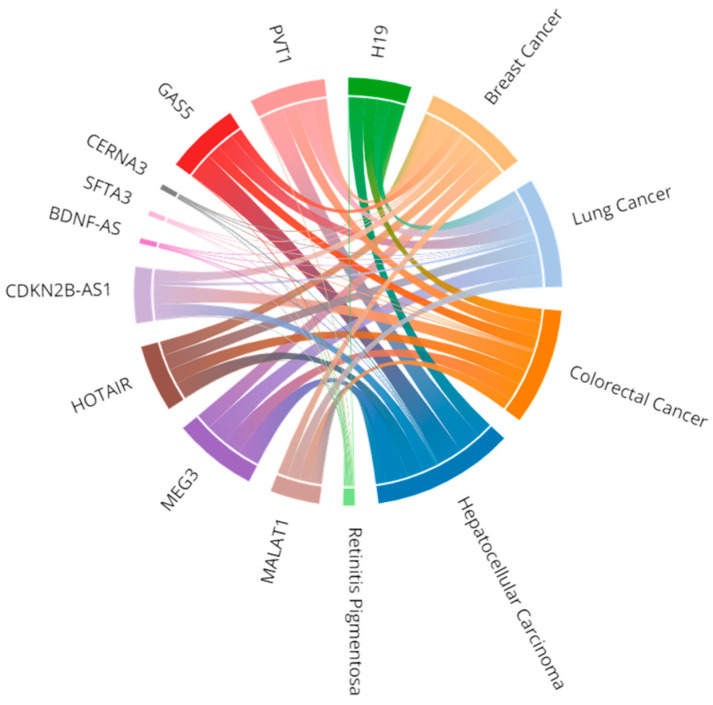
A map of gene-to-disease associations. Chord diagram of the network of the top 10 most-recurring genes, and the top 5 most-recurring diseases. Each chord represents the gene/disease association, where the width of the line correlates with the association score. The association score follows the MalaCards gene to disease scoring system, where score values depend on the level of manual curation of the information source, and on the significance assigned by the source itself to its different annotation classes [21].

Similarly, out of 2019 diseases, 1395 diseases were associated with only one gene (Figure 3b). The top five diseases with the most genes are gastric cancer (120 genes), retinitis pigmentosa (118 genes), hepatocellular carcinoma (117 genes), colorectal cancer (114 genes) and lung cancer (111 genes). A total of 1401 diseases with 3630 associations were purely inferred, out of which 1047 were non-cancerous diseases, e.g., autism spectrum disorder (22 gene associations), and 354 were cancerous diseases, for example, gastric cancer (120 associations). Moreover, only nine diseases were found to have four or more elite disease-gene associations—featured in Figure 3c. Around 50% of these genes are associated with diseases in many-to-many relationships, and the connectivity of selected cases of gene-to-disease associations is shown in Figure 4. 

**Figure 5:**
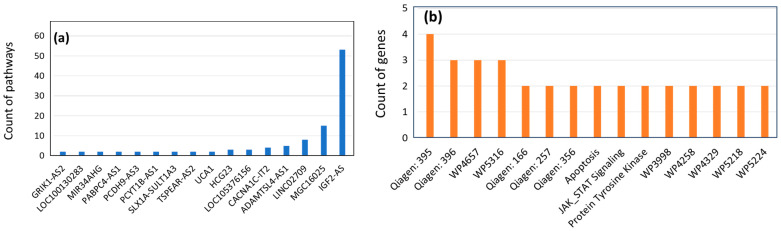

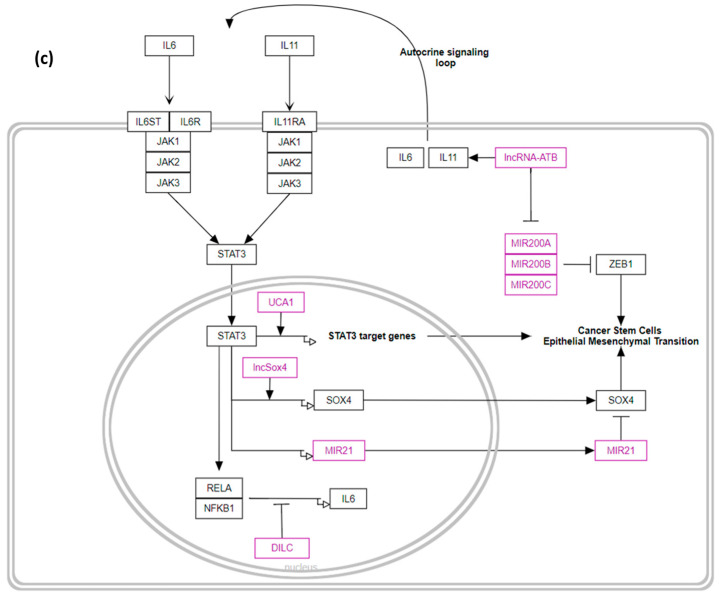
LncRNA-related pathways. (**a**) The counts of pathways (≥2) mapped to lncRNA genes. (**b**) The count of genes per pathway. 96 pathways include 1 gene (Table S4) and, at maximum, a pathway had 4 genes. (**c**) A map of the “STAT3 signaling in hepatocellular carcinoma” obtained from WikiPathways (ID: WP4337). The lncRNA genes involved represent the cruciality of lncRNA genes in regulating protein-coding genes, joining five MirRNA genes in the ncRNA category.

The involvement of multiple genes in a disease may indicate a gene–gene network, i.e., a biological pathway. Hence, all lncRNA genes associated with one or more diseases were analyzed with respect to their pathway affiliation, as portrayed in the Pathways section of GeneCaRNA, which is based on the PathCards database in the GeneCards suite [20]. In total, 131 pathways were found to be associated with 38 lncRNA genes (Figure 5a,b and Table S4). As an example, the role of four lncRNA genes in a pathway with 28 genes is shown (Figure 5c). 

### 3.4. Gene Product Interactions

**Figure 6:**
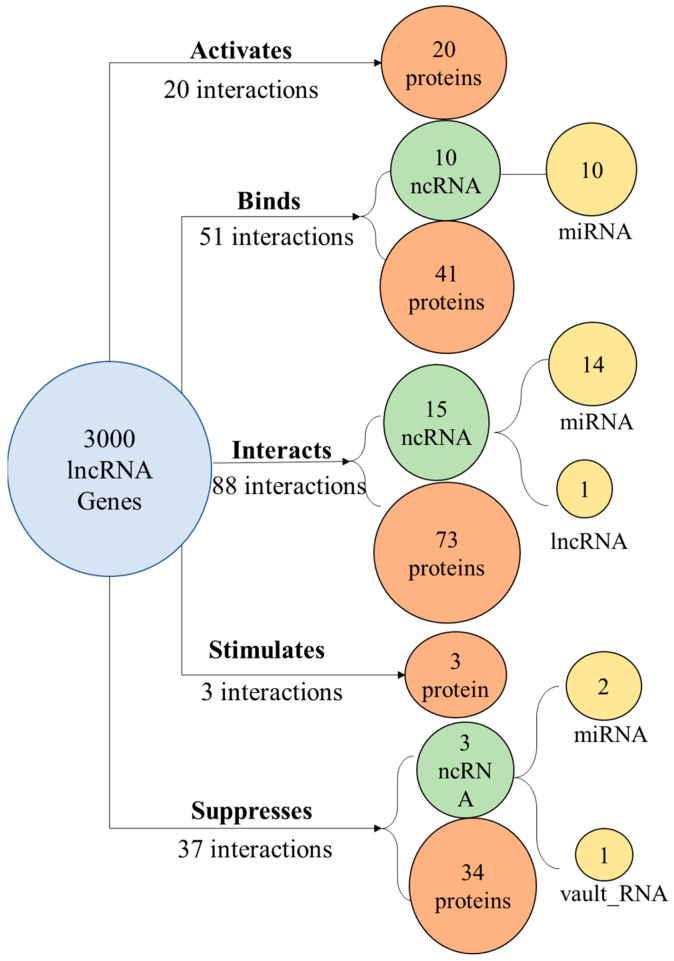
A diagram showing a sample of 199 target gene products showing one-to-one interactions with 118 lncRNA transcripts. The lncRNA targets include 171 proteins and a range of ncRNAs spanning three classes.

Our multiple gene text analysis tool (MuSe) was applied to obtain a partial image of interactions of lncRNA transcripts with transcripts and encoded proteins of other genes. This was performed with 3000 adequately annotated lncRNA genes using five interaction terms. This facilitated the identification of 199 interacting partners mapped to 118 genes. These partners included 28 ncRNA genes and 171 protein/protein-coding genes (Figure 6). A sample of these interactions (Table S5) is shown in more detail in Table 1, showing the broad range of ncRNA interactions.

Another method for finding functional relations is offered by GeneHancer, yet another facility of the GeneCards Suite [22]. This resource portrays regulatory elements (enhancers and promoters, collectively coined GeneHancers) for every gene in GeneCards, including all ncRNA genes. GeneHancer also provides a list of genes sharing enhancer(s) with the gene in question. It also exhibits potential phenotypes and diseases based on genome-wide association studies (GWAS), as shown in Figure S1. We extracted enhancer information for each LncRNA gene and found ~108,000 genes having at least one GeneHancer mapped to it due to gene/GeneHancer proximity (Figure S2). In total, 19,105 of the genes were contributed via major gene sources, with ~11% having a one-to-one relationship; 88,974 were TRIGGs, with ~17% of them in one-to-one relationships. Importantly, the very large group of novel TRIGG gene definitions often have no annotations except for those inferred via GeneHancer, opening a way for a massive body of functional-leading information. 

## 4. Discussion

GeneCaRNA was established three years ago to provide a compendium of all definable genes stemming from a full list of human transcripts. Its annotation system benefits from the fact that GeneCaRNA is part of the GeneCards Suite, which incorporates data mined from 194 sources [16]. Our database succeeds in portraying as many as ~280,000 ncRNA genes, approximately seven times more than several other resources. This significant improvement was attained by our TRIGG algorithm [16]. A great majority of the transcripts leading to the novel ~240,000 genes are weakly annotated, but the richness of their hosting GeneCards Suite often provides new information for such genes. In this paper, we use this power to scrutinize the largest class of ncRNA genes, namely lncRNAs, a functionally diverse group of genes, which currently embodies an avalanche of new research. GeneCaRNA, encompassing ~120,000 lncRNA genes, 43% of all ncRNA genes, provides substantial enrichment compared to other sources. Our GeneCaRNA-based enhancing pipeline for data extraction and analysis provides a fruitful approach to reporting and augmenting the relevant published information. 

The immense size and functional diversity of the lncRNA class calls for subclassification. The HUGO gene nomenclature committee (HGNC) defines six subclasses based on 6000 lncRNAs [1]. On the other hand, GENCODE defines nine subclasses based on 18,000 genes [11]. When analyzing all 120,000 of GeneCaRNA’s genes, we ascribed five subclasses to ~11,000 genes, of which ~600 were TRIGGS. We are now in the process of integrating GENCODE’s subclass assignments for 8000 genes not yet covered by GeneCaRNA. All of our five subclasses appear in other subclassification systems. Note that our subclass “Protein suspect”, a seeming contradiction to the definition of lncRNA [23], is a merger of “3′ overlapping” and “Sense overlapping” in GENCODE. We elected not to include HGNC’s “contain microRNA or snoRNA” and GENCODE’s “macro lncRNA”, “non_coding”, and “processed_transcript” lncRNAs. 

The lncRNA category is a functionally diverse class of ncRNAs [24,25]; a few lncRNA subclasses share functional attributes with other ncRNA classes, such as antisense lncRNAs, that affects protein-coding genes by regulating the fate of mRNAs, like miRNAs. On the other hand, a lncRNA subclass like Protein suspect is unique regarding its potential to encode polypeptides that are yet to be researched. In the case of the STAT3 signaling pathway (Figure 5c), where the exact mode of action of UCA1, a Protein suspect lncRNA, is uncertain [26], it could end up being explained by a novel encoded protein. 

The compilation of 11 data sources in PathCards, another GeneCards Suite member [20], successfully compiled information about 225 ncRNA genes of all classes, assigning each to one or more pathways. Of these, only 38 lncRNAs are associated with pathways. The capacities of the GeneCards Suite will surely support a significant enhancement in this field.

The study of lncRNAs is at the forefront of RNA biology, due to the rise in reports claiming the role of lncRNAs in human disorders [27]. A key strength of GeneCaRNA is its inclusion in a framework that has profound information about diseases via the inter-database relations with our MalaCards [21] suite member. MalaCards contains ~20,000 ncRNA gene–disease associations, of which 5100 associations are for LncRNA genes. This computes to 43 diseases per thousand genes in this class. We note that the gene-centric characteristics of GeneCaRNA are crucial for seeking new information on disease-gene because they facilitate the future assignment of genomic variations to diseases. This will become even more important due to the current capacities of the lncRNA gene class. The lack of gene–disease association in lncRNAs encourages utilizing the powerful capacities of GeneCaRNA in clinical studies, thus likely increasing the knowledge of disease-related lncRNAs. This could help bring the levels of lncRNA to that of miRNAs, which have 2119 diseases per thousand genes. 

In parallel, there are mechanisms to discover indirect relationships between lncRNA genes and diseases via GeneHancer (Figure S2) [22]. Regarding the distinction between elite and inferred gene–disease associations, while the former is based on solid experimental support, the latter often provides a hint that opens a gateway for more research, which may improve our understanding of a relevant disease. 

A key capability of GeneCaRNA is text analyses on gene annotations, revealing information on interactions between ncRNA transcripts and other gene products (protein and transcripts). Using this platform, we were able to portray a partial view of such an interaction network (Table S5). Further database development will allow us to obtain a more holistic picture. Some examples of currently missing data include published functional roles of lncRNAs in the context of inflammatory diseases and cancers [28,29]. This happens when lncRNAs bind to miRNAs, thus blocking their regulation of mRNAs in the translation of downstream genes. Broadening the lncRNA interactome in GeneCaRNA will strengthen its capacity to support future research in the field. 

The GeneCards suite also includes VarElect [16,30], which can connect DNA variants to diseases. Initially, it was developed for exome sequencing, by its ability to map variants in protein-coding genes to diseases and/or phenotypes. The establishment of GeneCaRNA now enables VarElect to also function in the realm of disease decipherment of variants in ncRNA genes obtained from whole genome sequencing. In particular, the indirect mode of VarElect helps discover the association of variants in ncRNA genes with a disease known to be caused by mutations in a protein-coding gene. This is achieved based on GeneCards information pointing to functional relationships between this ncRNA and the protein gene. This is important for solving undeciphered disease cases and is an essential instrument for expanding the knowledge of the mechanisms by which lncRNA gene mutations lead to diseases. For example, somatic copy number mutations in lncRNA genes are shown to be associated with cancer clinical prognosis [31], where, at present, little is known about the molecular pathways involved. 

In summary, GeneCaRNA provides a comprehensive, unified repository of lncRNA and its annotative information. Exploiting the forte of the GeneCards Suite, it is slated to become a powerful go-to place for researchers to explore these genes. The proposed text analysis pipeline for revealing gene–gene interactions, to be integrated into the GeneCards platform, will likely improve the understanding of the role of lncRNAs in biological processes. The realm of the TRIGGS and inferred gene–disease associations provides crucial terra incognita for pathobiology research, especially for poorly understood diseases. As knowledge of lncRNAs continues to evolve, the frequently updating GeneCaRNA will serve as an essential platform, enabling researchers to navigate the expansive landscape of non-coding RNA, including lncRNAs, their targets, and their associated regulatory routes, offering significant contributions towards disease decipherment. 

## Figures and Tables

**Table 1:** List of lncRNA genes and their associated target gene products.

S.no	Input LncRNA Genes		Action (Keyword)	Target Genes			
	Symbol	Description		Symbol	Description	Category	Class
1	BCAR4	Breast Cancer Anti-Estrogen Resistance 4	Activates	GLI2	GLI Family Zinc Finger 2	Protein-Coding	transcription regulator
2	HOTAIR	HOX Transcript Antisense RNA	Activates	DNMT1	DNA Methyltransferase 1	Protein-Coding	methyltransferase
3	HOXA11-AS	HOXA11 Antisense RNA	Activates	ITGB3	Integrin Subunit Beta 3	Protein-Coding	receptor
4	UCA1	Urothelial Cancer Associated 1	Activates	MTOR	Mechanistic Target Of Rapamycin Kinase	Protein-Coding	kinase
5	SNHG1	Small Nucleolar RNA Host Gene 1	Activates	HOXA1	Homeobox A1	Protein-Coding	transcription factor
6	LINC00261	Long Intergenic Non-Protein Coding RNA 261	Binds	MIR8485	MicroRNA 8485	RNA Gene	miRNA
7	ZFAS1	ZNFX1 Antisense RNA 1	Binds	MIR548E	MicroRNA 548e	RNA Gene	miRNA
8	H19	H19 Imprinted Maternally Expressed Transcript	Binds	EZH2	Enhancer Of Zeste 2 Polycomb Repressive Complex 2 Subunit	Protein-Coding	enzyme
9	HOTAIR	HOX Transcript Antisense RNA	Binds	AR	Androgen Receptor	Protein- Coding	receptor
10	LINC01413	Long Intergenic Non-Protein Coding RNA 1413	Binds	HNRNPK	Heterogeneous Nuclear Ribonucleoprotein K	Protein- Coding	nuclear ribonucleoprotein
11	NORAD	Non-Coding RNA Activated by DNA Damage	Binds	WEE1	WEE1 G2 Checkpoint Kinase	Protein- Coding	kinase
12	MIR222HG	MiR222/221 Cluster Host Gene	Interacts	DNM3OS	DNM3 Opposite Strand/Antisense RNA	RNA Gene	lncRNA
13	SNHG16	Small Nucleolar RNA Host Gene 16	Interacts	MIR195	MicroRNA 195	RNA Gene	miRNA
14	FOXD2-AS1	FOXD2 Adjacent Opposite Strand RNA 1	Interacts	EZH2	Enhancer Of Zeste 2 Polycomb Repressive Complex 2 Subunit	Protein- Coding	enzyme
15	CASC11	Cancer Susceptibility 11	Interacts	HNRNPK	Heterogeneous Nuclear Ribonucleoprotein K	Protein- Coding	Nuclear Ribonucleoprotein
16	DSCAM-AS1	DSCAM Antisense RNA 1	Interacts	YBX1	Y-Box Binding Protein 1	Protein- Coding	RNA-binding protein
17	HOTAIR	HOX Transcript Antisense RNA	Interacts	AR	Androgen Receptor	Protein- Coding	receptor
18	CRNDE	Colorectal Neoplasia Differentially Expressed	Interacts	TLR3	Toll Like Receptor 3	Protein- Coding	receptor
19	PCAT1	Prostate Cancer Associated Transcript 1	Interacts	AR	Androgen Receptor	Protein- Coding	receptor
20	RMST	Rhabdomyosarcoma 2 Associated Transcript	Interacts	SOX2	SRY-Box Transcription Factor 2	Protein- Coding	transcription factor
21	H19	H19 Imprinted Maternally Expressed Transcript	Stimulates	STAR	Steroidogenic Acute Regulatory Protein	Protein- Coding	regulator of steroid hormone synthesis
22	HULC	Hepatocellular Carcinoma Up-Regulated Long Non-Coding RNA	Stimulates	EMT	EMT ITK (IL2 Inducible T Cell Kinase)	Protein- Coding	kinase
23	SNHG8	Small Nucleolar RNA Host Gene 8	Stimulates	NPC	NPC (Intracellular Cholesterol Transporter)	Protein- Coding	transporter
24	MALAT1	Metastasis-Associated Lung Adenocarcinoma Transcript 1	Suppresses	MIR211	MicroRNA 211	RNA Gene	miRNA
25	SNHG1	Small Nucleolar RNA Host Gene 1	Suppresses	SOX9	SRY-Box Transcription Factor 9	Protein- Coding	transcription factor

## Data Availability

Use of GeneCards Suite websites is free for academic non-profit institutions. The suite’s extensive knowledge base is available for research purposes via an academic collaboration agreement (see https://www.genecards.org/guide/datasets (accessed on 3 June 2024)), Other users must acquire a commercial license from LifeMap Sciences.

## Supplementary Materials

The following supporting information can be downloaded at: https://www.mdpi.com/article/10.3390/biomedicines12061305/s1, Figure S1: Exhibits GeneHancer table; Figure S2: Rank graph to represent the distribution of number of enhancers per lncRNA gene; Table S1: List of all lncRNA genes in GeneCaRNA; Table S2: List of genes mapped to five subclasses; Table S3: List of elite gene–disease associations; Table S4: List of gene-to-pathway associations; Table S5: List of gene-to-gene interactions for five selected keywords.

## Abbreviations

LncRNA	Long non-coding RNA
ncRNA	Non-coding RNA
HGNC	HUGO Gene Nomenclature Committee
NCBI	National Center for Biotechnology Information
TRIGGs	Transcripts Inferred GeneCaRNA genes.
SQL	Structured Query Language
GIFtS	GeneCards Inferred Functionality Scores
LincRNA	Long intergenic non-coding RNA
MuSe	Multigene Search
ORF	Open Reading Frame
PE	Protein Evidence
